# Co-Culture with *Listeria monocytogenes* within a Dual-Species Biofilm Community Strongly Increases Resistance of *Pseudomonas putida* to Benzalkonium Chloride 

**DOI:** 10.1371/journal.pone.0077276

**Published:** 2013-10-10

**Authors:** Efstathios Giaouris, Nikos Chorianopoulos, Agapi Doulgeraki, George-John Nychas

**Affiliations:** 1 Department of Food Science and Nutrition, University of the Aegean, Myrina, Lemnos island, Greece; 2 Veterinary Research Institute of Athens, Greek Agricultural Organization “Demeter”, Aghia Paraskeui, Greece; 3 Department of Food Science and Human Nutrition, Laboratory of Microbiology and Biotechnology of Foods, Agricultural University of Athens (AUA), Athens, Greece; University of Birmingham, United Kingdom

## Abstract

Biofilm formation is a phenomenon occurring almost wherever microorganisms and surfaces exist in close proximity. This study aimed to evaluate the possible influence of bacterial interactions on the ability of *Listeria monocytogenes* and *Pseudomonas putida* to develop a dual-species biofilm community on stainless steel (SS), as well as on the subsequent resistance of their sessile cells to benzalkonium chloride (BC) used in inadequate (sub-lethal) concentration (50 ppm). The possible progressive adaptability of mixed-culture biofilms to BC was also investigated. To accomplish these, 3 strains per species were left to develop mixed-culture biofilms on SS coupons, incubated in daily renewable growth medium for a total period of 10 days, under either mono- or dual-species conditions. Each day, biofilm cells were exposed to disinfection treatment. Results revealed that the simultaneous presence of *L. monocytogenes* strongly increased the resistance of *P. putida* biofilm cells to BC, while culture conditions (mono-/dual-species) did not seem to significantly influence the resistance of *L. monocytogenes* biofilm cells. BC mainly killed *L. monocytogenes* cells when this was applied against the dual-species sessile community during the whole incubation period, despite the fact that from the 2nd day this community was mainly composed (>90%) of *P. putida* cells. No obvious adaptation to BC was observed in either *L. monocytogenes* or *P. putida* biofilm cells. Pulsed field gel electrophoresis (PFGE) analysis showed that the different strains behaved differently with regard to biofilm formation and antimicrobial resistance. Such knowledge on the physiological behavior of mixed-culture biofilms could provide the information necessary to control their formation.

## Introduction

Biofilms are commonly defined as communities of microorganisms attached to a surface or interface and producing an extracellular matrix, in which these microorganisms are embedded [1,2]. In the food industry, biofilm formation by spoilage and pathogenic bacteria has been of considerable interest and has provoked the interest of many research groups [3]. Obviously, the attachment of such bacteria onto food-contact surfaces and the subsequent biofilm formation is undesirable, since the detachment of cells from the biofilm structure can lead to the cross-contamination of the food products, causing food spoilage and / or foodborne diseases [4-6]. The risk becomes even more serious, since it has been observed that the antimicrobial resistance of biofilm cells is significantly increased compared to planktonic ones [7]. 

In food processing environments, a variety of different bacteria are known to attach to surfaces, survive, grow and form sessile biofilm communities [8-16]. Spatial and metabolic interactions between species are believed to contribute to the organization of multispecies biofilms, and the production of a dynamic local environment [17-20]. Mixed-species biofilms are usually more stable than mono-species biofilms, while cell-to-cell interactions and communication have been demonstrated to play a key role in initial cell attachment, biofilm growth and structure, cell dispersion, as well as in the resistance of biofilm community members against antimicrobial treatments [21-38]. 

*Listeria monocytogenes* is a ubiquitous Gram-positive facultative intracellular bacterial pathogen capable of surviving and growing under a wide range of adverse environmental conditions, such as high acidity, high salinity and at refrigeration temperatures [39,40]. It is the causal agent of listeriosis, a severe foodborne disease which provoked 134 human deaths in European Union in 2011, with a reported high case fatality ratio of 12.7% [41]. Notably, many *L. monocytogenes* strains are capable of adhering to various both biotic (e.g. animal tissues) and abiotic (e.g. stainless steel, plastic) surfaces and create biofilms (for a review see 42). Attachment to surfaces is believed to be important for survival and persistence of this pathogen in food processing environments, with some strains being able to remain on equipment surfaces even for several years [43-46].

*Pseudomonas* spp. are Gram-negative obligatory aerobic bacteria, capable of degrading a variety of low molecular weight organic components and are very common in fresh foods, mainly because of their widespread existence in water, soil and vegetation. Many *Pseudomonas* species are psychrotrophic and therefore important spoilage microorganisms of raw foods stored under refrigeration [47-49]. Pseudomonads are among the better-studied microorganisms with respect to phenotypic changes taking place throughout the process of biofilm formation and the genetic determinants involved [50-53]. These are commonly found in the food-processing environment [54,55] and have been previously documented as good producers of extracellular polymeric substances, including polysaccharides, nucleic acids, and proteins [56-58], making them ideal organisms with which to investigate the growth of *L. monocytogenes* within multispecies biofilms. Notably, *P. putida* bacteria are capable of adhering to various food contact surfaces and form strong biofilms [5,53,59-62]. This species is also known to produce biosurfactants, amphipathic molecules which have been shown able to influence biofilm development and moreover to break down existing biofilms [63,64].

Benzalkonium chloride (BC) is a biocide belonging to the group of quaternary ammonium compounds (QACs) that are commonly used in both food and medical environments [65,66]. QACs possess antimicrobial effect against a broad range of microorganisms, since they act on general membrane permeability, causing cytolytic leakage of cytoplasmic material at low concentrations. At high concentrations, they target the carboxylic groups and cause general coagulation in the bacterial cytoplasm [67,68]. However, the frequent use combined with the misuse of disinfectants, such as BC, in food environments can lead to the development of cellular adaptation mechanisms and the emergence of disinfectant resistant cells [69-74]. As thus, resistance to QACs has been reported in many Gram positive as well as Gram negative bacteria associated with food [75,76]. 

In recent years, the study of mixed species sessile communities composed of various foodborne bacteria has increased the understanding of interactions and dynamics of surface attached bacteria and biofilms under conditions relevant to food processing [23,25,35,61,77-79]. Evidently, multi-species biofilms are dynamic communities with extensive interactions taking place between the different species and strains [17,80], which probably have a significant effect on the structure, composition (extracellular matrix constituents), population dynamics (i.e. which species and / or strain is present / dominates) and physiology (i.e. function, metabolism, resistance, virulence) of mature communities. Particularly challenging is the attempt to understand the complexity of all these interactions encountered within such communities, and how this may influence the final community outcome and behavior. 

To this direction, in this study, some selected *L. monocytogenes* and *P. putida* strains (three strains per species) were left to form biofilms on stainless steel (SS) surfaces, under either mono- or dual-species conditions, at 18 °C, for a total period of 10 days, with daily medium renewal. Both types of biofilms (mono-/dual-species) were daily subjected to chemical disinfection by applying inadequate (sublethal) concentration of BC (50 ppm). The microbial composition of the mono-species sessile communities the first day of incubation, as well as of the dual-species community the last day of incubation, with regard to strain occurrence, just before and following disinfection, was also evaluated by using a pulsed field gel electrophoresis (PFGE) approach. Results obtained highlight the impact of bacterial interactions taking place inside such mixed-culture sessile communities on both their population dynamics and chemical disinfection resistance and could be helpful in our efforts to control mixed-culture biofilm formation by unwanted bacteria in food processing areas.

## Materials and Methods

### Bacterial strains and growth conditions

Three *L. monocytogenes* (FMCC_B-125, FMCC_B-129, FMCC_B-169) and three *P. putida* (CK119, CK120, CK148) strains, isolated from different origins, were used in this study. Regarding the *L. monocytogenes* strains, FMCC_B-125 was the clinical reference strain ScottA (serotype 4b, lineage I, epidemic strain, human isolate, [81]), originally supplied by the Agrotechnological Research Institute ATO-DLO (Wageningen, the Netherlands), FMCC_B-129 was isolated from a ready-to-eat minced meat based frozen meal [25], and FMCC_B-169 was isolated from the environment of an Italian food processing plant (strain 2UD of DSA collection, [82]). The selection of the three *L. monocytogenes* strains was based on previous comparative results of biofilm formation on polystyrene microplates by 11 *L. monocytogenes* strains in total under different growth conditions [25]. Regarding the *P. putida* strains, all were isolated from minced beef stored at 5 °C [48].

Before utilization, all the microorganisms were stored frozen (at -80°C) in bead vials (Protect; Technical Service Consultants Ltd, Heywood, Lancashire, UK) and each one was then resuscitated by adding one bead to 100 ml of Brain Heart Infusion (BHI) broth (LAB M; International Diagnostics Group Plc, Bury, Lancashire, UK) in a conical flask and incubating at 30°C for 24 h under agitation (precultures). Working cultures were prepared by adding a 100-μl aliquot of each preculture to 100 ml of BHI broth and incubating at 30°C for 16 h (under agitation), at which time late exponential phase was attained for each strain (data not shown). Cells from final working cultures were harvested by centrifugation (5000 x *g*, 10 min, at 4 °C), washed twice with ¼ Ringer solution (Ringer’s tablets; Merck, Darmstadt, Germany) and finally resuspended in ¼ Ringer solution (*ca.* 10^9^ CFU ml^-1^), in order to be used as inocula for the biofilm development assays. 

### Abiotic substratum and biofilm formation procedure

Stainless steel (SS) coupons (3 x 1 x 0.1 cm, type AISI-304, Halyvourgiki Inc., Athens, Greece) were the abiotic substrates used for biofilm formation, since this material is frequently used for the manufacture of food processing equipment. Prior to use, coupons were cleaned according to the procedure described by Kostaki et al. [25]. Following cleaning, coupons were individually placed in empty glass test tubes (length, 10 cm; diameter, 1.5 cm) and autoclaved at 121°C for 15 min.

To produce biofilms on SS coupons, three strains per species were selected and left to produce biofilms, under either mono- or dual-species conditions, according to the protocol described by Kostaki et al. [25]. Briefly, two subsequent steps were followed: sterile coupons were initially incubated in saline bacterial suspensions (bacterial attachment step), and afterwards coupons carrying strongly attached bacteria were incubated in daily renewable growth medium (biofilm formation step).

 For the attachment step, 5 ml of bacterial suspension in ¼ Ringer solution, containing *ca*. 10^8^ CFU ml^-1^ for each strain, was poured into each one glass test tube containing a sterilized SS coupon, followed by incubation at 18°C for 3 h, under static conditions. Care was taken in order the bacterial suspension to contain approximately the same number of cells for each strain (*ca*. 10^8^ CFU ml^-1^). 

Following attachment step, each coupon was carefully removed from glass test tube using sterile forceps and was thereafter rinsed by immersing it, for 5 min, in 5 ml of ¼ Ringer solution, with shaking, in order to remove the loosely attached cells. After rinsing, coupon was individually introduced in new sterile glass test tube containing 5 ml of Tryptone Soy Broth (TSB; LAB M) and subsequently incubated at 18°C for a total period of 10 days (240 h), under static conditions, to allow biofilm development on the coupon. Growth medium was renewed every 24 h. During each medium renewal, loosely attached cells were removed by rinsing (as described above).

### Exposure of biofilm cells to disinfection

Every 24 hours, each SS coupon – carrying biofilm cells onto it – was carefully removed from glass test tube using sterile forceps and was thereafter rinsed by pipetting two times 10 ml of ¼ Ringer solution (each time), in order to remove the loosely attached cells. “Strong twice pipetting” was chosen here as a more harsh method to do this, compared to “immersing with shaking” which was previously followed (following the initial 3 h attachment step and daily in the course of biofilm formation). After this rinsing, coupon was individually introduced in new glass test tube containing 5 ml of 50 ppm of benzalkonium chloride (BC) solution. BC was purchased from Sigma-Aldrich (Life Science Chemilab S.A., Athens, Greece) and its desired solution was prepared in distilled water. Before use for disinfection treatments, BC was checked for sterility by agar plating. Disinfection was carried out at 18°C for 6 min to imitate disinfection conditions encountered in real food processing areas.

### Recovery and quantification of viable biofilm cells

On 1^st^, 2^nd^, 4^th^, 6^th^, 8^th^ and 10^th^ day of incubation, the viable biofilm bacteria on SS coupons, just before and following the 6-min exposure to BC disinfection, were quantified by using the “bead vortexing method” described by Kostaki et al. [25], in which strong vortexing of each coupon with glass beads is used to detach the biofilm cells. It should be noted that following BC disinfection, SS coupons were incubated for 10 min in Dey-Engley neutralizing broth in order to stop BC action and also help viable stressed / injured cells to recover. Detached cells were subsequently enumerated by agar plating, after ten-fold serial dilutions. In the case of mono-species biofilm development, Tryptone Soy Agar (TSA; LAB M) was used for the enumeration of bacteria. In the case of dual-species biofilm development, the cells of each one species were enumerated using the following selective media: PALCAM *Listeria* Selective agar (PALCAM; LAB M) for *L. monocytogenes* and *Pseudomonas* Agar (CFC Agar; LAB M) for *P. putida*. Previously, it had been confirmed that *P. putida* cells were not able to grow on PALCAM plates, while *L. monocytogenes* cells were not able to grow on CFC plates. Additionally, TSA was also used in the case of dual-species biofilm monitoring to have a comparison with the results obtained by the selective media (results not shown). In all cases, plates were incubated at 30°C for 48 - 72 h. 

### Isolation of strains from mixed-culture biofilm communities

In order to monitor the individual contribution of each *L. monocytogenes* and *P. putida* strain in both the development and resistance of mixed-culture (mono-/dual-species) biofilm communities, 12 colonies were randomly selected from the highest dilutions of agar plates (used to enumerate the viable bacteria recovered from SS coupons by the bead vortexing method just before and following disinfection) the first and the last day of incubation. In particular, out of a total number of 96 colonies picked, half of them (48) were recovered from the mono-species biofilms the 1^st^ day of incubation (containing either *L. monocytogenes* or *P. putida* strains), while the other half (48) were recovered from the dual-species biofilm the 10^th^ day of incubation (containing both *L. monocytogenes* and *P. putida* strains). All isolated colonies were stored at -80°C in TSB containing 20% (v/v) glycerol (Serva GmbH, Heidelberg, Germany), until PFGE analysis. 

### Pulsed-field gel electrophoresis (PFGE) analysis

PFGE of *L. monocytogenes* strains was performed according to Kostaki et al. [25], while the PFGE protocol described by Doulgeraki and Nychas [48] was followed for the *P. putida* strains. 

### Statistical analysis

Each experiment included three replicate coupons and was repeated twice using independent bacterial cultures. Microbiological counts were transformed to logarithms before means and standard deviations were computed. All data were analyzed by analysis of variance (ANOVA) by the general linear model procedure of SPSS data analysis software for Windows (release 10.0.1; SPSS Inc., Chicago, IL). Least square means were separated by Fisher’s least significant difference (LSD) test. All differences are reported at a significance level of an alpha of 0.05.

## Results

### Effect of culture conditions (mono-/dual-species) on biofilm formation by *L. monocytogenes* and *P. putida* strains

**Figure 1:**
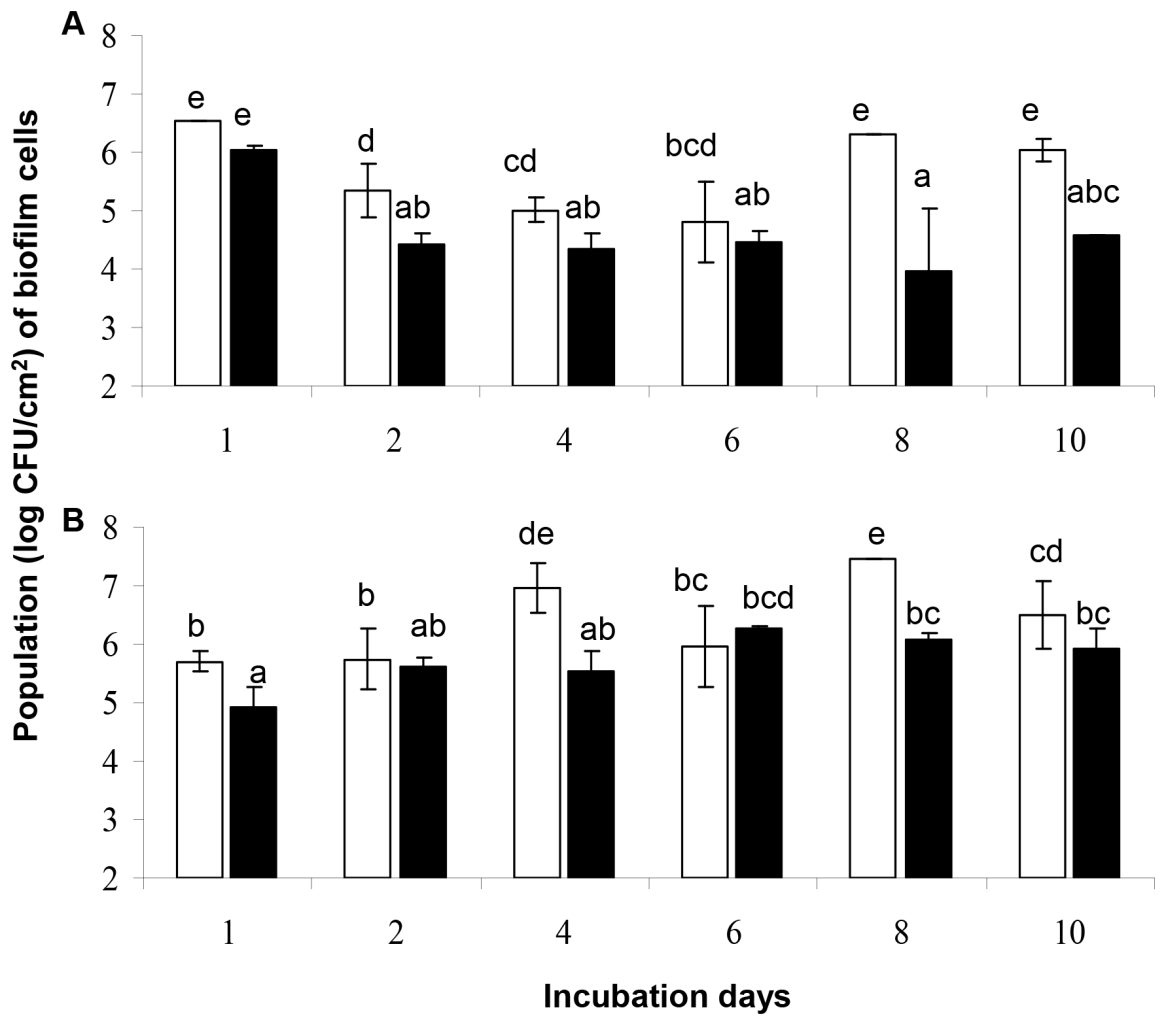
Populations (log CFU/cm^2^) of biofilm cells on SS coupons, just before disinfection. (A) *L. monocytogenes*; (B) *P. putida*. Biofilms were left to be formed on coupons incubated at 18 °C for a total period of 10 days in daily renewable TSB, under either mono-species (□, three strains of each one species together), or dual-species conditions (■, six strains of the two different species together) and subjected daily to disinfection (6-min exposure to 50 ppm of BC solution). The bars represent the mean values ± standard deviations (n=6, two independent experiments, each performed three times). For each graph separately, mean values sharing at least one common lower case letter shown above the bars are not significantly different at a *P* value of <0.05.

The results regarding the populations (log CFU/cm^2^) of *L. monocytogenes* and *P. putida* biofilm cells on SS coupons, at the different incubation (sampling) days, just before the disinfection treatment, either under mono- or dual-species conditions, are presented in Figure 1. In general, it is observed that the simultaneous presence of both bacterial species in the biofilm community seems to lead to a reduction of their sessile populations, compared to when each species was left to form biofilm separately from the other.

Regarding *L. monocytogenes* (Figure 1A), culture conditions (mono-/dual-species) significantly (*p* < 0.05) influenced its final (after 10 days) biofilm population level. Thus, under mono-species conditions, *L. monocytogenes* reached a sessile population of 6.05 log CFU/cm^2^, while under dual-species conditions, this population was approximately 30 times less (4.57 log CFU/cm^2^). Besides the 10^th^ day of incubation, the simultaneous presence of *P. putida* cells also significantly reduced the population of *L. monocytogenes* biofilm cells the 2^nd^, 4^th^ and 8^th^ day of incubation (fold difference ranged from 4.5 times less the 4^th^ day to more than 234 times less the 8^th^ day). Rather strangely, the 1^st^ and 6^th^ day of incubation, *L. monocytogenes* biofilm counts did not significantly differ between mono- and dual-species conditions. 

Regarding *P. putida* (Figure 1B), culture conditions (mono-/dual-species) do not seem to significantly influence its final (after 10 days) biofilm population level. Thus, this bacterium reached final sessile populations of 6.50 and 5.93 log CFU/cm^2^ for mono- and dual-species conditions, respectively. However, the simultaneous presence of *L. monocytogenes* cells significantly (*p* < 0.05) reduced the population of *P. putida* biofilm cells the 1^st^, 4^th^ and 8^th^ day of incubation. For instance, the 8^th^ day, *P. putida* reached under mono-species conditions a sessile population of 7.46 log CFU/cm^2^, while under dual-species conditions, this population was more than 20 times less (6.09 log CFU/cm^2^). 

### Effect of culture conditions (mono-/dual-species) on BC resistance of *L. monocytogenes* and *P. putida* biofilm cells

**Figure 2:**
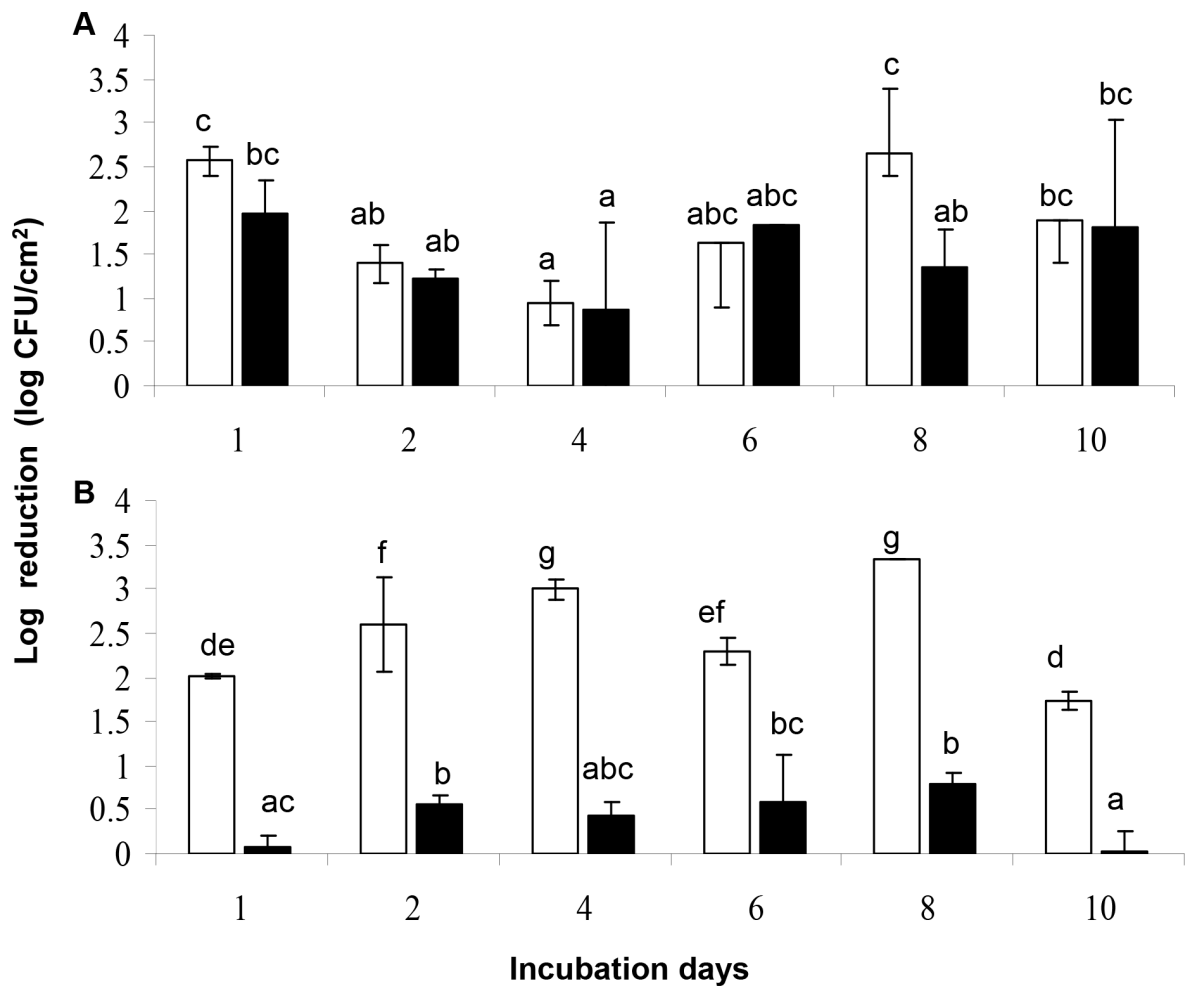
Log reductions (log CFU/cm^2^) of biofilm cells on SS coupons, following disinfection. (A) *L. monocytogenes*; (B) *P. putida*. Biofilms were initially left to be formed on coupons incubated at 18 °C for a total period of 10 days in daily renewable TSB, under either mono-species (□, three strains of each one species together), or dual-species conditions (■, six strains of the two different species together) and subjected daily to disinfection (6-min exposure to 50 ppm of BC solution). The bars represent the mean values ± standard deviations (n=6, two independent experiments, each performed three times). For each graph separately, mean values sharing at least one common lower case letter shown above the bars are not significantly different at a *P* value of <0.05.

The effect of the 6-min disinfection treatment with 50 ppm of BC solution on *L. monocytogenes* and *P. putida* biofilm cells, cultured under either mono- or dual-species conditions, was expressed as biofilm population log-reduction (difference in log CFU/cm^2^ values just before and after the treatment) (Figure 2). 

Regarding *L. monocytogenes* (Figure 2A), it was observed that, in general, culture conditions (mono-/dual-species) do not seem to significantly influence the resistance of its biofilm cells against BC (except the 8^th^ day of incubation). Thus, for instance, the last (10^th^) day of incubation, log reductions of 1.89 and 1.82 log CFU/cm^2^ were monitored for mono- and dual-species conditions, respectively. Under current experimental setup, this bacterium presented the higher susceptibility the 8^th^ day under mono-species conditions (log reduction 2.64 log CFU/cm^2^), while the most resistance was recorded on the 4^th^ day (log reductions of 0.94 and 0.88 for mono- and dual-species conditions, respectively). 

Regarding *P. putida* (Figure 2B), culture conditions (mono-/dual-species) significantly influenced the resistance of its biofilm cells against BC. In particular, the simultaneous presence of *L. monocytogenes* cells led to a significant increase of the resistance of *P. putida* biofilm cells to the chemical disinfection during whole incubation period. Thus, while under mono-species conditions log reduction of *P. putida* cells ranged from 1.74 log CFU/cm^2^ (the 10^th^ day) to 3.33 log CFU/cm^2^ (the 8^th^ day), under dual-species conditions the highest log reduction recorded was just 0.79 log CFU/cm^2^ (the 8^th^ day). 

### Microbial (species) composition of the dual-species biofilm communities

**Figure 3:**
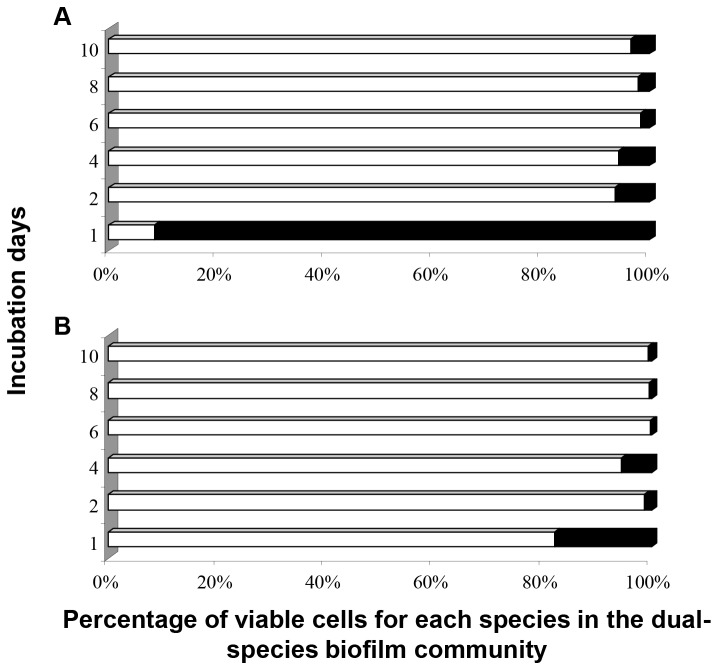
Percentages of viable *L. monocytogenes* (filled bars) and *P. putida* (open bars) cells in the dual-species biofilms. (A) Just before disinfection; (B) Following disinfection. Dual-species biofilms were initially left to be formed on SS coupons incubated at 18 °C for a total period of 10 days in daily renewable TSB and subjected daily to disinfection (6-min exposure to 50 ppm of BC solution).

The results on the microbial (species) composition of the dual-species biofilm communities formed on SS coupons, at the different incubation (sampling) days, for *L. monocytogenes* and *P. putida* cells, just before and following the 6-min exposure to 50 ppm of BC solution are presented in Figure 3. 

**Figure 4:**
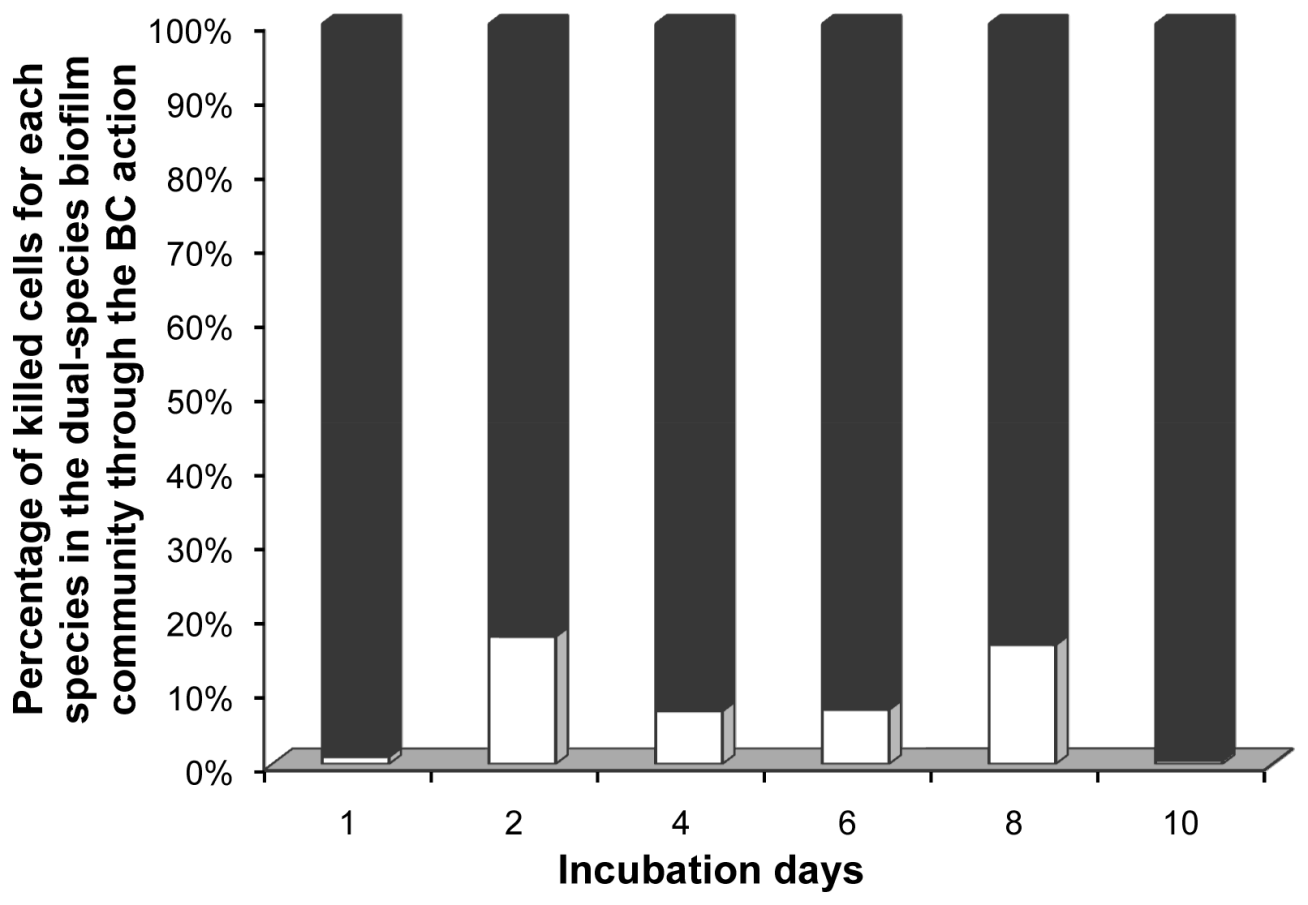
Percentages of killed *L. monocytogenes* (filled bars) and *P. putida* (open bars) cells in the dual-species biofilms. Dual-species biofilms were initially left to be formed on SS coupons incubated at 18 °C for a total period of 10 days in daily renewable TSB and subjected daily to disinfection (6-min exposure to 50 ppm of BC solution).

According to these results, while *L. monocytogenes* dominated in the dual-species sessile community the 1^st^ day of incubation (91.6% out of the total biofilm cells belonged to it), all other next days the dual-species community was mainly composed of *P. putida* cells (>90%) (Figure 3A). In the same way, for each sampling day, following disinfection, the dual-species community was mainly composed of *P. putida* cells. Thus, the percentage of viable *P. putida* cells out of the total number of biofilm cells ranged from 82.3% the 1^st^ day to 99.9% the 6^th^ day (Figure 3B). Interestingly, under current applied experimental setup, BC mainly killed *L. monocytogenes* biofilm cells, when this was applied against the dual-species biofilm communities, during the whole incubation period (Figure 4). Thus, the percentage of killed *L. monocytogenes* cells out of the total number of killed cells ranged from 82.8% the 2^nd^ day to 99.5% the 10^th^ day (Figure 4).

### Distribution of *L. monocytogenes* and *P. putida* strains in the mixed-culture biofilm communities

**Figure 5:**
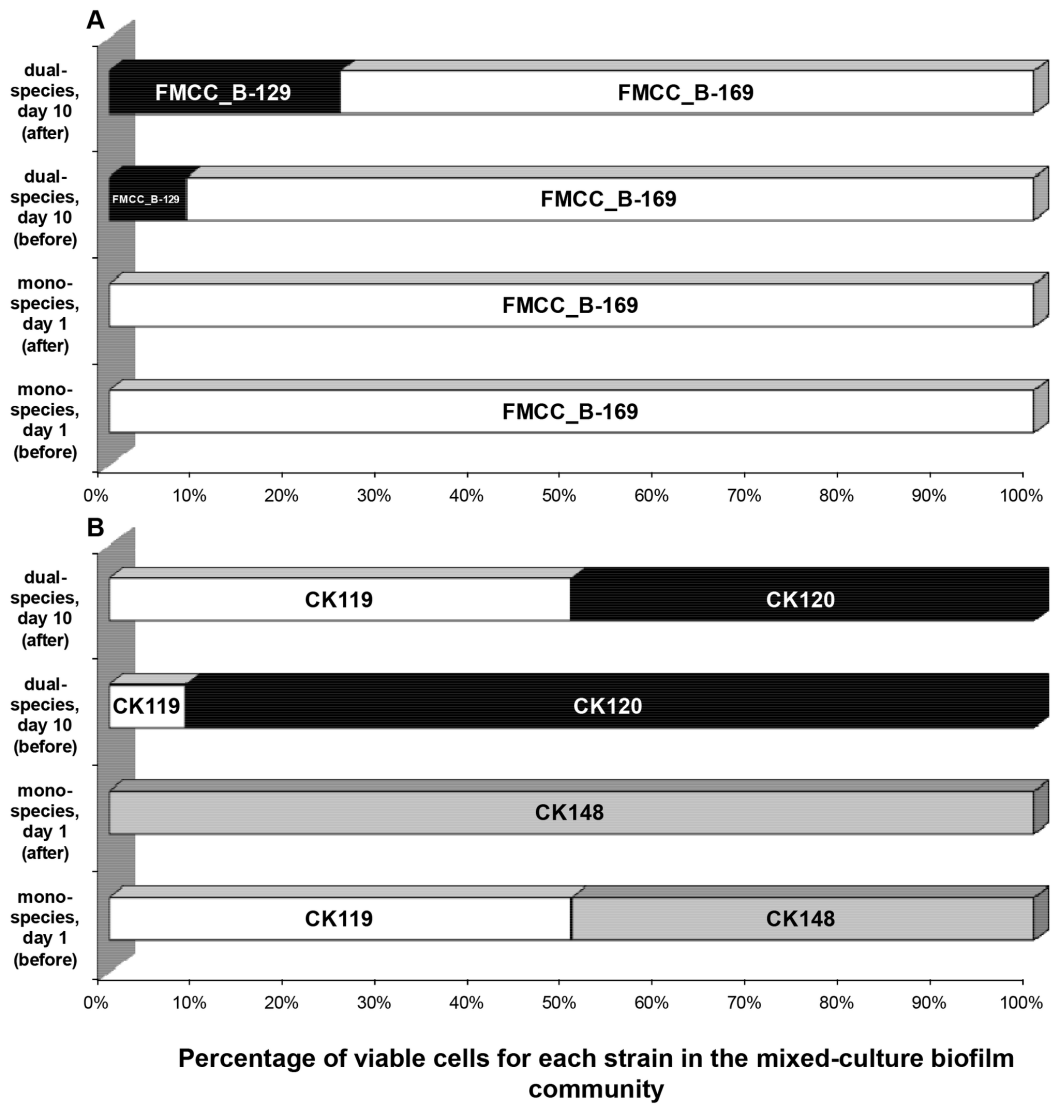
Percentages of viable cells for each strain in the mixed-culture biofilm communities. (A) *L. monocytogenes*; (B) *P. putida*. Mixed-culture (mono-/dual-species) biofilms were left to be formed on SS coupons incubated at 18 °C for a total period of 10 days in daily renewable TSB and subjected daily to disinfection (6-min exposure to 50 ppm of BC solution). Graphs present the distribution of viable strains in the mono-species biofilm communities (the 1^st^ day of incubation), as well as in the dual-species biofilm community (the 10^th^ day of incubation), just before and after disinfection.

The individual contribution of each *L. monocytogenes* strain (FMCC_B-125, FMCC_B-129, FMCC_B-169) and each *P. putida* strain (CK119, CK120, CK148) in the composition of the mono-species biofilm communities (the 1^st^ day of incubation), as well as in the dual-species biofilm community (the 10^th^ day of incubation), just before and after the 6-min exposure to 50 ppm of BC solution is illustrated in Figure 5.

According to these results, it is obvious that the different strains employed here (3 strains / species) did not contribute at the same levels to either the formation of these mixed-culture sessile communities or their antimicrobial recalcitrance. Regarding *L. monocytogenes* (Figure 5A), FMCC_B-169 strain, originally isolated from the environment of a food processing plant (strain 2UD of DSA collection, [82]), was found to dominate in all biofilm communities tested. Thus, under mono-species conditions, this strain completely dominated from the 1^st^ day of incubation. Under dual-species conditions (the 10^th^ day), this strain consisted the 91.7% of the total fraction of *L. monocytogenes* biofilm cells before the disinfection treatment. This percentage was reduced to 75% following disinfection. In the latter case, the other 25% belonged to FMCC_B-129 strain, originally isolated from a ready-to-eat frozen meal [25]. It’s worth to be noted that the clinical FMCC_B-125 (ScottA) strain, originally isolated from human [81], seemed to be unable to develop biofilm on SS coupons, under current experimental conditions and sampling days. 

Regarding *P. putida* (Figure 5B), CK119 and CK148 strains equally contributed to the formation of the mono-species biofilm community the 1^st^ day of incubation, while the CK120 strain seemed to be unable to compete the other two strains in the course of biofilm formation. Following disinfection, the mono-species biofilm community composed exclusively of the CK148 strain, which proved to be more resistant to the BC antimicrobial action. On the contrary, under dual-species conditions, CK148 strain was totally absent the 10^th^ day of incubation. In the latter case and with regard to *P. putida* cells, dual-species community was composed by 91.7% of CK120 strain, while the other 8.3% belonged to CK119 strain. Following disinfection, dual-species community was equally composed by the CK119 and CK120 strains. 

## Discussion

In the majority of natural and man-made environments, microorganisms usually associate with surfaces in complex multi-species communities [83-87]. The structural and functional dynamics of multi-species biofilms are largely due to the interactions between the different species [17,31,88-90]. However, this complexity is not taken into consideration when growing microorganisms in monocultures under laboratory conditions. In this study, the simultaneous biofilm formation by six selected *L. monocytogenes* and *P. putida* strains was investigated (3 strains per species). These were left to develop mixed-culture biofilms on SS coupons incubated in daily renewable growth medium, while biofilm communities were also, in daily basis, subjected to inadequate (sub-lethal) disinfection treatment with 50 ppm of BC solution. “Glass bead vortexing” was used to dislodge biofilm cells for counting onto selective media, since it has been previously proven effective to give “rough” estimations of biofilm counts [25,61]. Although this method may not be effective to completely remove all the biofilm cells from the surface, it still gives quite more accurate results compared to other methods employed for this purpose (e.g. swabbing, sonication). Undoubtedly, the possibility to use in parallel a sensitive microscopic technique for obtaining high-resolution optical images of the formed biofilm (e.g. by using different fluorescent probes for the two different species and observing them under confocal laser scanning microscopy, CLSM) would be a valuable help in our effort to unravel the microbial (species) composition of the dual-species biofilm community. 

Obtained results (Figure 1) revealed that under such conditions, both bacteria were able to develop a dual-species biofilm, in which however *P. putida* was found to dominate from the 2^nd^ day of incubation (Figure 3). Thus more than 90% out of the total number of biofilm cells belonged to this species. The observed dominance of *P. putida* over *L. monocytogenes* was something rather expected given the well-known ability of Pseudomonads, including *P. putida*, to produce a variety of extracellular polymeric substances (EPS, e.g. cellulose, alginate, Pel and PsI exopolysaccharides), which help them to form strong biofilm communities either on abiotic or even biotic surfaces [50,51,58,59,62,91-93]. The increased tolerance to BC of the Gram-negative *P. putida* compared to the Gram-positive *L. monocytogenes* may also account for this observation. Additionally, *P. putida*, together with other species of the same genus, is also known to produce biosurfactants [63,94], well-known anti-biofilm compounds which have been shown to inhibit both attachment and biofilm formation by other species or even disperse already established biofilms [64,95-97]. 

On the contrary, the foodborne pathogen *L. monocytogenes* does not always have a high potential for forming strong mono-species biofilms *in vitro* on food contact materials at relevant food industry conditions [98-102], but surfaces already colonized by other bacteria may significantly increase its adherence and biofilm formation [103-105]. In this context, Hassan et al. [104] noticed, when studied the behaviour of *L. monocytogenes* on condensate-covered stainless steel with a *P. putida* biofilm over a total period of 35 days, that *L. monocytogenes* attached in significantly greater numbers (> 3-log difference) to surfaces with pre-existing *P. putida* biofilms than to *Pseudomonas*-free surfaces. In another study and in accordance with current results, Chorianopoulos et al. [61] found, when co-cultured on SS surfaces 5 strains belonging to *Salmonella enterica*, *L. monocytogenes*, *P. putida*, *Staphylococcus simulans* and *Lactobacillus fermentum*, that mixed-culture biofilm was mainly composed of *P. putida* cells (97.8%), while *S. enterica* and *L. monocytogenes* represented together only the 2.2% of it. Similarly, Lourenço et al. [106] observed, when evaluated biofilm formation by 4 selected *L. monocytogenes* strains (at 12 and 37 °C) either on pure cultures or on co-cultures with *P. aeruginosa* (PAO1), that in co-culture biofilms, *P. aeruginosa* was the dominant species, at both temperatures, representing 99% of the total biofilm population. 

In a number of previously published studies, attachment and biofilm formation by *L. monocytogenes* and *P. putida* have been shown to be influenced by either the natural *in situ* presence of other species or just their metabolic by-products [10,29,36-38,55,61,103,104,107-113]. For instance, the presence of *Staphylococcus xylosus* and *Pseudomonas fragi* affected the numbers of *L. monocytogenes* biofilm cells on stainless steel [113], while Carpentier and Chassaing [10] reported that among 29 tested dairy environmental strains, 53% and 13%, reduced or enhanced *L. monocytogenes* biofilm formation, respectively. In the present study, it was in general observed that the simultaneous presence of both bacterial species in the dual-species biofilm community seems to lead to a reduction of their sessile populations, compared to mono-species conditions (Figure 1). It’s worth to be noted that each one of the six strains employed here was also screened for inhibitory activity against the other strains using the well diffusion assay. However, such activity was not revealed (data not shown). Likewise, Norwood and Gilmour [113] found, when determined the differential adherence capabilities at three different temperatures of two *L. monocytogenes* strains to SS by submerging stainless steel coupons in both 48-h *Listeria* monocultures and mixed cultures additionally containing *Staphylococcus xylosus* and *P. fragi*, that the monoculture biofilms consistently contained greater *L. monocytogenes* numbers than the multispecies biofilms.

In recent years, several studies have also been conducted on the resistance of mixed species biofilms to disinfectants [25,35,61,78,111,112,114-117]. However, most of these relevant studies performed did not include results of single species biofilm resistance, making it impossible to judge whether there is any effect of interspecies interactions on the resistance of each individual species in the mixed community. In a recent study, Saá Ibusquiza et al. [78] studied the resistance to BC and the microscopic structure between mixed-species biofilms formed by four different strains of *L. monocytogenes* and one strain of *P. putida* under different scenarios. They found that the presence of *P. putida* in *L. monocytogenes* biofilms quickened biofilm formation and significantly increased their resistance to BC with respect to the resistance of mono-species *L. monocytogenes* biofilms after 4 days of incubation at 25 °C. These authors suggested that the resistance of mixed-species biofilms of *L. monocytogenes* and *P. putida* to BC seems to be related to their microscopic structure and to the association between the involved species. On the contrary, in the present study, culture conditions (mono-/dual-species) did not seem to significantly influence the resistance of *L. monocytogenes* biofilm cells to BC, while these had a profound effect on the resistance of *P. putida* cells (Figure 2). In particular, it was observed that the simultaneous presence of *L. monocytogenes* strongly increased resistance of *P. putida* biofilm cells to BC. It should be noted that the effect of the 6-min disinfection treatment on biofilm cells was expressed as biofilm population log-reduction (difference in log CFU/cm^2^ values just before and after the treatment) in order to take also into account the initial biofilm counts (before disinfection). In a recent relevant study of dual-species biofilm formation between three *L. monocytogenes* and three *S. enterica* strains, interspecies interactions did not significantly influence neither the biofilm forming ability, nor the antimicrobial resistance of each individual species [25].

Contradictory literature results on biofilm research data are probably not solely explained by the inherent differences between the different strains, but also by the fact that the interactions encountered in mixed-culture biofilm communities depend on a number of factors, such as nutritional conditions, bacterial co-aggregation, metabolic requirements, exposure to antimicrobial agents and other environmental factors (e.g. shear forces, temperatures, atmosphere etc) [118]. Any change in these factors can drastically impact the structure, dynamics and thus the behaviour of the biofilm community [84,90]. Obviously, the above examples emphasize the principle that studies on biofilm formation by foodborne bacteria should be performed under relevant mixed-culture conditions employing a variety of different strains and species. Additionally, special care should be taken when educing conclusions based on the results of a single study, since biofilms seem to be very diverse and unique, not just to the microorganism, but to the particular environment in which they are being formed. 

Under current applied experimental conditions, it was observed that BC mainly killed *L. monocytogenes* biofilm cells, when this was applied against the dual-species biofilm communities, during the whole incubation period (Figure 4). This was rather expected given that it is well known that Gram negative bacteria are less susceptible to QACs than Gram positive bacteria, and additionally *Pseudomonas* spp. have generally high intrinsic resistance compared with other Gram negative bacteria [72,119]. However, it is still quite interesting that the percentage of killed *L. monocytogenes* cells out of the total number of killed cells exceeded 80% (Figure 4), in dual-species communities containing more that 90% of *P. putida* cells (Figure 3). Interactions leading to specific spatial distribution of cells having different resistance to disinfectant in mixed-species biofilms may also explain observed resistance of *P. putida* biofilm cells [20,26,108]. By applying different disinfectants, Fatemi and Frank [116] investigated the ability of peracetic acid and peroctanoic acid sanitizers to inactivate mixed-culture biofilms of a *Pseudomonas* sp. and *L. monocytogenes* on SS and they found that *Pseudomonas* and *L. monocytogenes* were inactivated to similar levels by the sanitizer treatments, even though *Pseudomonas* predominated in the initial biofilm population.

It is generally acknowledged that microbial resistance to disinfectants may develop following exposure to sublethal concentrations of them [73,120]. Thus, many studies have demonstrated that bacteria, including *L. monocytogenes* and pseudomonads, are capable of adapting to disinfectants used in industrial settings after prolonged exposure to sublethal concentrations [75,76,121,122]. BC-resistance among *L. monocytogenes* strains isolated from food sources can vary from 10% [123] to over 40% [124,125], while more than 30% of the pseudomonads isolated from poultry carcasses were found able to grow in the presence of BC at the concentrations used in poultry plants [126]. However, in the present study no obvious adaptation to BC was observed in either *L. monocytogenes* or *P. putida* biofilm cells, since log reductions, consequence of the 6-min exposure to 50 ppm of BC solution, did not significantly differ between first and last day of incubation, for both growth conditions (mono-/dual-species) (Figure 2). In a biofilm study examining the morphological and biochemical changes in *P. fluorescens* biofilms grown in the presence of subinhibitory concentrations of 4 antimicrobial agents including BC, it was observed that *P. fluorescens* exhibited adaptation to BC at 10 mg / ml [69]. In another study examining biofilm formation by 95 *L. monocytogenes* strains and also aiming to determine the extent to which biofilm production protects this pathogen against QAC challenge (50 or 150 ppm for 60 sec), it was concluded that it is the maturity of the biofilm, rather than the strain itself, which is actually correlated with QAC resistance [100].

In current study, a promising PFGE approach was also used to monitor the individual contribution of each *L. monocytogenes* and *P. putida* strain in the formation and maintenance of mono- and dual-species biofilm communities, whether or not these were exposed to BC disinfection. In order to achieve this, two time periods in the course of biofilm development were selected; one at the first day of incubation and the other at the last (10^th^) day. Results revealed that different strains behave differently with regard to their ability to develop mixed-culture biofilms and their antimicrobial recalcitrance (Figure 5). Under mono-species conditions, from the 1^st^ day of incubation, only one strain for each species dominates (this is FMCC_B-169 for *L. monocytogenes* and CK148 for *P. putida*). As expected, this situation was found to remain the same until the end of incubation (10^th^ day) (results not shown). The higher initial attachment ability, the higher specific growth rate and / or the better entrapment ability in the developing biofilm structure (and as thus released dispersal), as well as the increased resistance to BC of some strains, compared to the other strains also being present, may explain why some strains were found to dominate in each mixed-culture biofilm community. However, it should be noted that the BC susceptibility of each individual strain (i.e. under mono-culture conditions) was on purpose chosen not to be examined, since under mixed-culture conditions, bacterial interactions may influence both biofilm formation ability and BC susceptibility of each individual strain. The last becomes more evident if we take into account the possible complex 3D biofilm structure which may develop when the different strains are left to develop biofilm together. Although under dual-species conditions the strain distribution could have been monitored each sampling day, this was examined only at last (10^th^) incubation day (mainly due to practical reasons). Despite this, it is still obvious that bacterial interactions among the different strains and species seem to have a significant influence on the growth potential, survival and more generally in the “individual behaviour” of each strain. In the same way, in a recently published study [25], both intra- and inter-species bacteria interactions encountered inside mono- and dual-species biofilms of *L. monocytogenes* and *S. enterica* were found to have a profound effect on both the population dynamics, as well as on the resistance pattern of each *L. monocytogenes* strain being present. Competitive interactions among *L. monocytogenes* strains in mixed-culture biofilms have also been previously observed between serotype 1/2a and 4b strains [127].

## Conclusions

In summary, present results highlight the impact of bacterial interactions taking place inside a mixed-culture sessile community on both its population dynamics and chemical disinfection resistance. Interestingly, under dual-species conditions, the simultaneous presence of *L. monocytogenes* strongly increased resistance of *P. putida* biofilm cells to BC. Following disinfection of dual-species sessile community with BC, the vast majority of cells killed belonged to *L. monocytogenes*, while the remaining viable community was mainly composed of *P. putida* cells (>90%). In general, under dual-species conditions, the simultaneous presence of both bacterial species led to a reduction of their sessile populations, compared to mono-species conditions. Additionally, besides the differences observed in the dual-species biofilms with regard to species occurrence, differences in strain dominance were also observed in mixed-culture biofilm communities. Different strains were found to dominate, according to surrounding environmental conditions. Obviously, the results of another relevant study on biofilm formation of these two species (under both mono- and dual-species conditions) without any disinfection treatment could help us to more clearly unravel the effect of culture conditions (mono-/dual-species) on the biofilm forming ability of each individual species and strain, and also to unravel the possible effect of biofilm maturation on biofilm composition. However, in this study, the conditions encountered in real food processing areas, that are daily disinfection of biofilm cells, were deliberately chosen.

Evidently, bacterial pathogens, such as *L. monocytogenes* and spoilage bacteria such as *P. putida*, can be entrapped in multi-species sessile communities formed on inadequately cleaned and disinfected food processing surfaces. However, in real food environments, the possible presence of many other microbial species clearly adds additional complexity to the behaviour of multi-species biofilms, since all incorporated microorganisms are able to compete, cooperate, and communicate with each other. Undoubtedly, further research is required to improve our understanding on the physiology of multi-species biofilms formed by food related bacteria. This could probably facilitate the development of methods for controlling them in food areas and therefore reduce the contamination of the food products.
